# The complex relationships among self-acceptance, perceived social support, drug use stereotype threat, and subthreshold depression in people with substance use disorder: exploring the mediating and buffering effects

**DOI:** 10.3389/fpsyt.2025.1444379

**Published:** 2025-02-03

**Authors:** Yongqiu Li, Rufang Wang, Jun Liu, Zuoliang Li, Yinghua Zhou

**Affiliations:** ^1^ School of Management, Chengdu University of Traditional Chinese Medicine, Chengdu, China; ^2^ School of Basic Medical Sciences, Chengdu University of Traditional Chinese Medicine, Chengdu, China; ^3^ Rehabilitation Department, Sichuan Drug Rehabilitation Administration, Chengdu, China; ^4^ Psychological Correction Center, Ziyang Drug Rehabilitation Center, Ziyang, China; ^5^ Psychological Correction Center, Chengdu No. 2 Drug Rehabilitation Center in Sichuan Province, Chengdu, China

**Keywords:** people with substance use disorder, self-acceptance, perceived social support, drug use stereotype threat, subthreshold depression

## Abstract

**Introduction:**

Depression levels are significantly higher among people with substance use disorder (SUD) than in the general population; however, studies on the level of subthreshold depression in this population are scarce. Research shows a significant correlation between self-acceptance and depression, with social support playing a key role in the process of recovery and social reintegration for people with SUD. This study aimed to explore the effects of self-acceptance, perceived social support, and stereotype threat of people with SUD on their subthreshold depression, as well as potential mediating and buffering effects.

**Methods:**

This study was conducted from January-March 2024. 1068 drug addicts (548 males and 520 females) were recruited in Chengdu, Sichuan Province. After signing informed consent, their psychometric data were obtained using the Self-acceptance Questionnaire (SAQ), Perceived Social Support Scale (PSSS), Drug Use Stereotype Threat Scale (DSTS) and Center for Epidemiologic Studies Depression Scale(CES-D). Gender and group differences in relevant scale dimensions were explored. Linear regression models were used to assess the relationships between PSSS, SAQ, and DSTS scores and subthreshold depression in male and female participants. Bootstrap mediation effect tests were used to further test the mediation effect of drug use stereotype threat and perceived social support between self-acceptance and subthreshold depression. Line graphs were used to show the buffering effect of perceived social support on the relationship between self-acceptance and subthreshold depression in different groups.

**Results:**

The results showed that,gender, HIV-positive or not, education and monthly income level affect subthreshold depression in patients with SUD. Negative correlation between self-acceptance and subthreshold depression among SUD patients. Furthermore, perceived social support and substance use stereotypes threatmediated the relationship between self-acceptance and subthreshold depression, respectively, forming a parallel mediating relationship. Results exploring the buffering effect of perceived social support by subgroup showed that the buffering effect of perceived social support on subthreshold depression was most pronounced in the HIV-negative and and female groups. Social support, group stereotypes discrimination affect the mental health of sud patients.

**Discussion:**

Our study provides theoretical support for the alleviation of subthreshold depression among people with SUD, realizing that self-acceptance, perceived social support and reduction of drug use stereotype threat can be a psychoprotective factor for people with SUD.

## Introduction

Substance use disorder (SUD) is a crucial issue worldwide. Data from the World Drug Report 2024 released by the United Nations Office on Drugs and Crime shows 292 million people globally will use drugs in 2022, 20% higher than 10 years ago (1). According to the China Drug Situation Report 2023 (2), there are now 896,000 drug addicts in China, a year-on-year decrease of 20.3 per cent, and 4,078,000 patients who have been abstinent for three years and have not relapsed, a year-on-year increase of 7.6 per cent; the number of abusers of the main popular drugs continues to decrease, and anti-drug efforts have achieved remarkable results. At the same time, however, some people with SUD have turned to new psychoactive substances, such as dextromethorphan, and other non-scheduled substances for substitution and abuse in order to alleviate their drug addiction. Several prospective studies have shown a clear link between anxiety and depression and the severity of SUD, with a high prevalence of anxiety and depression among people with SUD (3). Many psychiatric disorders are associated with an increased risk of late-stage SUD conditions (4), and subthreshold depression is linked to an increased risk of developing major depressive disorder (5). Long-term drug use gives people with SUD negative and negative social identities and social evaluations, which are accompanied by emotions of self-depreciation and self-appraisal, affecting the process of their detoxification and rehabilitation. Thus, the impact of subthreshold depression on the mental health of people with SUD cannot be ignored. For SUD patients, irrational beliefs were significantly higher for men than for women (5), a result that implies that gender differences may play an important role in the psychological mechanisms behind drug use behaviors; the psychological energy of the jobless was significantly lower than that of the employed, and the higher the income, the higher the level of psychological energy (6), implying that SUD patients of lower socioeconomic status are at a higher risk for abnormalities in their mental health status. Therefore, whether gender and economic income are the demographic factors affecting subthreshold depression in SUD patients is worth further exploring. People with both SUD and HIV comprise a specific group for which the stigma associated with both HIV and SUD can have a negative impact on individual health (7). People with SUD who are HIV-positive may face substance use and HIV stigma that complicates their livelihoods, health status, and participation in care (8). Thus, paying attention to the mental health status of this group is essential. For HIV-positive SUD patients, exploring which variables affect their subthreshold depression is of positive significance for subsequent targeted formulation of intervention strategies to improve their mental health.

### Self-acceptance and Subthreshold Depression

Self-acceptance is the ability of an individual to assess his or her own goals and turn them into reality, that is, the individual compares his or her own qualities with those of others, receives the opinions of others about him or her, and uses these opinions as a reference for his or her own enrichment and modification (9). Self-acceptance is considered vital to an individual’s mental health (10, 11). Individuals with high levels of self-acceptance typically exhibit greater self-confidence and hold more positive emotional attitudes toward themselves. In contrast, people with low levels of self-acceptance are more likely to experience low self-esteem, introversion, and social anxiety (12). Subthreshold depression, also known as subclinical, is a psychological state characterized by prolonged negative mood, loss of interest, self-blame, low self-esteem, lack of attention, and other major symptoms of depression with no specific cause that is below the level necessary to meet the diagnostic criteria for depression (13). Several previous studies have been conducted on the depressive state of people with SUD, finding significantly higher levels of depression in this group than in the general population (14). Relatively few studies on subthreshold depression have been conducted with people with SUD as participants. Individuals with subthreshold depression share several similarities with people with major depression, and the prevalence of subthreshold depression is approximately twice as high as that of major depression (15). Moreover, individuals with subthreshold depression are at increased risk of developing major depression (16). Therefore, focusing on subthreshold depression among people with SUD may help to identify those at risk for major depression and prevent the deterioration of their mental state.

Emotion regulation theory emphasizes how individuals manage and adjust their emotional states to seek internal self-balance (17). Effective emotion regulation strategies can help individuals reduce the impact of negative emotions and improve mental health (18, 19). Self-acceptance can be viewed as an effective emotional regulation strategy in the addiction recovery process. When people with SUD are able to accept themselves for their past and current situation, they are more likely to adopt positive coping styles to deal with life’s challenges and difficulties. Conversely, if SUD patients are unable to accept themselves, they may be more likely to fall into negative patterns of thinking and behavior, leading to psychological imbalances. Empirical studies have shown that self-acceptance has been found to be a protective factor in mental health (10–12, 20), suggesting that it may also be a protective factor in subthreshold depression. From an empirical perspective, revealing the potential relationship between self-acceptance and subthreshold depression in patients with SUD is of great significance for updating information on designing specific treatments and interventions to reduce the likelihood of subthreshold depression in individuals with SUD and promote their better social adaptation.

Based on the theoretical and empirical literature, we are expected to formulate the first hypothesis of this study:

**H1. self-acceptance has a significant negative direct effect on subthreshold depression;**

### Self-acceptance, drug use stereotype threat, perceived social support and subthreshold depression

Steele and Aronson define a “stereotype threat” as the process of negative experiences that occur when an individual or a group perceives a negative stereotype during a negative experience for fear of corroborating the negative stereotype (21), while Inzlicht and Schmader consider stereotype threat to be a situational and social influence that exposes individuals to risks beyond their objective level of competence (22). The stereotype threat process model conceptualizes stereotype threat as a stage of imbalance in the interrelationships between a person’s relevant competencies, domains, and self-concept; when this imbalance occurs, stereotype threat arises (23). In summary, as Nie has posited, drug use stereotype threat can be defined as the feeling of threat that arises when they are aware of certain negative labels or when others evaluate them using negative labels (24). The higher the individual’s sense of discrimination, the lower the level of self-acceptance (25). The level of self-acceptance of people with SUD was significantly lower than the norm (26). Social identity theory states that individuals gain self-identity and self-esteem by classifying themselves into a certain social group. People with SUD are often excluded by the mainstream society due to the particularity of their behavior and identity. This long-term marginalization, negative social identity and social evaluation are accompanied by individual self-depreciation and self-stigma (27), which affects the process of rehabilitation of people with SUD (28). Stereotype threat is a self-validating fear that others and even oneself will evaluate one’s behavior on the basis of negative stereotypes. This triggers negative emotions, which may accumulate, affecting an individual’s mental health and social adaptation (29, 30).

Based on the theoretical and empirical literature, it is rational to propose the second hypothesis of the current study

**H2. Drug use stereotype mediates the associations between self-acceptance and subthreshold depression;**

Perceived social support refers to an individuals’ subjective perceptions and evaluations of their emotional experiences and the level of support, understanding, and respect they feel they receive from society (31). High levels of social support and low levels of social prejudice can improve the affective state of people with SUD (32). Studies have also suggested that for every 1-unit increase in family support, the probability of high levels of depressive symptoms decreases by 4.7% (33). Thus, obtaining social support may be positively associated with decreased subthreshold depression. Early establishment of social support is conducive to not only the reduction of dependence and relapse behaviors among people with SUD but also the improvement of their quality of life, thus facilitating their ability to maintain their recovery and reintegrate into society (34).

It has been noted that self-acceptance is not only significantly and positively related to self-esteem, it is a significant predictor of self-esteem (35). Perceived social support is positively correlated with self-esteem in people with SUD (36, 37). This implies that there may be some correlation between self-acceptance and perceived social support. In China, especially in highly traditional social settings, substance use disorders are widely viewed as an anti-social and cultural norm rather than merely a health issue (38). This deep-rooted cultural and ethical beliefs have led SUD populations to appreciate more social rejection, exclusion, and discrimination, and less often to experience positive social support. For SUD patients, higher levels of perceived social support can reduce their stress levels (39), increase their abstinence self-efficacy (40). Most importantly, numerous studies have found that high levels of perceived social support have a positive impact on individuals’ ability to improve their subjective well-being and maintain mental health (41–43). Given the findings from theoretical and empirical studies, it is expected to propose the hypothesis:

**H3. Perceived social support mediates the associations between self-acceptance and subthreshold depression;**

In addition, it should be noted that drug use stereotype and perceived social support are not independent mediators. People with a history of substance abuse are subject to widespread stigmatization. Such social opposition seems likely to lead to a sense of threat from stereotypes, or a perception that a person is the target of devaluing stereotypes. Stereotype threats have the potential to cause social functioning difficulties, including adverse social outcomes (44). Therefore, we proposed the fourth hypothesis regarding serial mediations:

**H4. The serial mediation path of self-acceptance→drug use stereotype→perceived social support→subthreshold depression is significant;**

### Self-acceptance, perceived social support, and subthreshold depression in SUD patients in different groupings

Self-acceptance can enhance the well-being of adolescents living with HIV (ALWHs) (45). Depressive symptoms are a risk factor for neuropsychological disorders in people who are HIV-positive (46),social support is considered an influential predictor of depression among people living with HIV (47, 48). Cross-sectional data on HIV-positive individuals who use intravenous drugs showed a significant negative association between their perceived social support and a significant reduction in depression levels (49). However, no research has proved that high-level perceived social support can reduce subthreshold depression caused by insufficient self-acceptance among HIV-positive drug addicts. Therefore, based on the empirical research, the following hypotheses are drawn:

**H5:Perceived social support may play as a buffer between self-acceptance and subthreshold depression in HIV-positive people with SUD**

Based on social role theory, male and female are given different role expectations and behavioral norms by society as they grow up. Male patients with SUD are subjected to greater stress (50). They follow the norms of gender roles for a long time, and tend to suppress their emotions when facing difficulties and challenges, and it is difficult to express their vulnerability and needs. Studies have shown that for women with SUD, quality of life is closely related to their perceived social support (51). Psychological functioning is included in quality of life. Higher social support leads to a higher level of mental health. Male SUD patients have higher levels of depression than women and have a more dangerous mental health state (14), and it is more difficult to intervene. Furthermore, women receive higher quality and level of social support than men (52, 53). Thus, based on the empirical research, the following hypotheses are drawn:

**H6:Perceived social support may play as a buffer between self-acceptance and subthreshold depression in women with SUD**

### The current study

To the best of our knowledge, although there are many studies on the relationship between self-acceptance and mental health, no study has explored the mediating and moderating roles of drug use stereotype threat and perceived social support, particularly in patients with SUD. First, demographic factors influencing subthreshold depression among individuals with SUD were explored; then, whether there were significant differences in relevant scale dimensions by gender and HIV-positive/negative subgroups was explored, and linear regression modeling was used to assess the relationship between PSSS, SAQ, and DSTS scores and subthreshold depression among male and female participants; and lastly, the present study explored the multiple factors potentially influencing subthreshold depression (self-acceptance, perceived social support, and drug use stereotype threat) whether there is a chain mediating role between self-acceptance, perceived social support, and subthreshold depression, and whether perceived social support serves as a buffer among HIV status and gender grouping.

## Materials and methods

### Participants and procedures

After ethical review by the Clinical Medical Research Ethics Committee of Ziyang People’s Hospital(2021-K-LS-2), this study was conducted from January–March 2024. The participants were informed of the purpose of their involvement in the program and provided signed informed consent before taking part in the survey. The Self-acceptance Scale (SAQ), Perceived Social Support Questionnaire (PSSS), Drug Use Stereotype Threat Questionnaire (DSTS), and Center for Epidemiologic Studies Depression Scale (CES-D) were administered by the professional staff (including three graduate students and one master tutor from Chengdu University of Traditional Chinese Medicine and the staff of the drug rehabilitation center). Participants provided the research data by completing the questionnaires and all participants received gifts as a token of appreciation.

This is a cross-sectional study, implemented in January-April 2024, at Ziyang Compulsory Drug Rehabilitation Center and Women’s Compulsory Drug Rehabilitation Center in Sichuan Province. The two drug rehabilitation centers in Ziyang and Deyang were chosen as the subjects of the study mainly based on their important position and representativeness in the anti-drug work in Sichuan Province. These two drug rehabilitation centers are relatively large in scale and can cover and reflect the situation of SUD patients from different backgrounds from all over Sichuan, which to a certain extent enhances the breadth and generalizability of the study results. This cross-sectional study could not only explore the current situation and relationships between variables in patients with SUD, but also explore whether the buffering effect of the variables was significant after grouping by HIV infection and gender.

Based on the principle of stratified whole-cluster sampling, drug addicts in the two compulsory isolation of people with SUD were tested in separate brigades. First, the number of people with SUD in each brigade within these two drug rehabilitation centers was sorted, and then the specific sample size of each brigade was determined by an equal proportion sampling strategy. Then, using Excel 2019, the corresponding random number intervals were set according to the specific range of the number of people in each brigade, and the people who were the subjects of the study were randomly selected by invoking the random function, and finally, the paper questionnaires were distributed to the participants under the on-site guidance of the professional investigators. The sample size based on reliability and validity should be 5–10 times or more than the number of items, therefore the number should be 290-580 or more (54). A total of 1072 questionnaires were distributed, 1072 questionnaires were recovered, and after excluding invalid questionnaires such as omissions, 1068 valid questionnaires were obtained (548 men and 520 women; 895 HIV-negative and 173 HIV-positive), with an effective rate of 99.6%.

The enrolment criteria for participants were as follows: (a) people with SUD who had completed physical detoxification and had a negative urine test, (b) met the DSM-V diagnostic criteria for abuse of or dependence on psychoactive substances, (c) did not have a severe psychiatric illness and were not taking medication, (d) were between 16–65 years of age, and (e) had an elementary school education or above.

### Instruments

#### Self-acceptance questionnaire

The SAQ (55) is a self-report scale for individuals to evaluate their self-acceptance and comprises two dimensions: self-appraisal and self-acceptance. Each dimension has 8 entries, which are rated using a 4-point Likert scale of “very incompatible,” “basically incompatible,” “basically compatible,” and “very compatible.” Items can receive scores ranging from 1 to 4, with total scores of 16 to 64 points. The higher the score, the higher the respondent’s self-acceptance level. In this study, the Cronbach’s Alpha value of SAQ was 0.708.

#### Perceived social support scale

The PSSS developed by Zimet et al. and revised by Yan et al. was adopted to determine the degree of perceived social support from various social support resources (56, 57). The scale is comprised includes 12 items across three dimensions: Family, Friends, and Significant Other. Each dimension has four items. The PSSS is scored on a 7-point Likert scale ranging from 1 (strongly disagree) to 7 (strongly agree), with higher scores reflecting higher levels of perceived social support. In this study, the Cronbach’s Alpha value of PSSS was 0.942.

#### Drug use stereotype threat scale

The DSTS is based on von Hippel’s Stereotype Threat Scale (44), which validates the applicability of the revised version of the scale to people with SUD in the Chinese cultural context (24). The scale consists of 10 total questions and 3 dimensions: stereotype awareness, stereotype identification, and alienation. Answers are provided using a 5-point Likert scale ranging from 1 (strongly disagree) to 5 (strongly agree). The higher the score, the stronger the substance use stereotype threat experienced by the respondent. In this study, the Cronbach’s Alpha value of DSTS was 0.885.

#### Center for epidemiologic studies depression scale

The CES-D, developed by Radloff (58), is the most commonly used representative primary screening tool for assessing subthreshold depression both nationally and internationally. It includes 20 questions in total and four dimensions: negative affect, positive affect, somatic complaints, and interpersonal difficulties (59). Responses are provided on the following 4-point Likert scale: 1 (occasionally or never: less than one day per week), 2 (sometimes: 1–2 days per week), 3 (often or half of the time: 3–4 days per week), and 4 (most of the time or persistent: 5–7 days per week). Total scores range between 0–60 points, with 16 points as the cutoff score indicating the likely presence of depressive symptoms. Higher scores indicate more severe depression. In this study, Cronbach’s alpha coefficient was 0.840.

### Statistical analysis

Excel,SPSS 25.0 and AMOS 24.0 were used for data entry and statistical analysis. Firstly, common method bias test was performed. Descriptive statistics were presented as M(SD)/Percent (%). Second, t-test, one-way ANOVA, and binary logistic regression were used to analyze whether there was a significant difference in subthreshold depression levels among SUD patients with different demographic variables. Then, participants were categorized into HIV-positive and HIV-negative groups, and means and standard deviations (M (SD)) were calculated for age, education, monthly income, and the dimensions of PSSS, CES-D, SAQ, and DSTS. It was investigated whether the variables were significantly different on grouping by gender and grouping by HIV status. Subsequently, correlations between variables were explored using heat map plots. Thirdly, SUD patients were grouped by gender and further explored what effect each variable had on the respective subthreshold depression of males and females. Mediation was then modeled using AMOS 24.0 to validate the concurrent mediating role of perceived social support and drug use stereotype threat, that is, to validate the significance of the pathway self-acceptance → drug use stereotype threat → perceived social support → subthreshold depression. Finally, the moderating role of perceived social support between self-acceptance and subthreshold depressive symptoms (between HIV-positive and HIV-negative groups and between male and female participants) was demonstrated using line plots.

## Results

### Common method bias test

We used a self-report questionnaire in this study; consequently, we performed principal component analysis (i.e., common method bias test) on all questionnaire items. A one-way factor analysis based on Harman, as suggested by Podsakoff (60), showed that 12 factors had eigenvalues greater than 1, and the maximum factor variance explained was 16.98% (less than 40%). Thus, common method bias was not a serious issue in this study.

### Sample description

Among the participants, methamphetamine use was the most predominant, accounting for 74.2%, while heroin use accounted for 32.6%. In addition to traditional drugs such as opium, marijuana, cocaine, and ecstasy, some participants also used new types of drugs, including nitrous oxide and etomidate. Most participants (89.9%) reported using one type of drug, while 11.1% reported the use of two or more. Furthermore, 60.8% of the participants had been using substances for 10 years or less, while 27.2% had used substances for 11–20 years, and 12% for more than 20 years.

Regarding the CES-D t-test and one-way ANOVA for demographic variables, **Table 1** shows a significant difference in subthreshold depression scores based on gender (p < 0.001), with women scoring higher than men. A statistically significant difference was also found in the degree of subthreshold depression between participants who were HIV-positive and those who were HIV-negative (p < 0.001), with a lower mean score for the HIV-positive group. Education level and monthly income were found to have significant within-group differences in influencing subthreshold depression (p < 0.001). After conducting a t-test and one-way ANOVA, covariate diagnosis was performed for variables with significant differences. The VIF values for gender, HIV status, education level, and monthly income were less than 2 (1.130–1.255), which indicated a lack of multiple covariance, allowing for binary logistic regression analysis.

**Table 1:** Independent sample t- and F-tests for demographic variables and binary logistic regression analysis of factors influencing subthreshold depression.

Characteristics		Percent(%)	M(SD)	*p (t/F)*	β	Standard error	Wald x^2^	*P*
Gender				<0.001	0.744	0.156	22.726	<0.001
	(Male)	51.31	1.01(0.48)					
	Female	48.69	1.21(0.46)					
HIV-positive				<0.001	0.316	0.204	2.415	0.120
	Yes	16.20	0.99(0.51)					
	(No)	83.80	1.13(0.47)					
Education level				<0.001				
	(Primary School)	21.3	1.24(0.44)		–	–	–	–
	Junior high school	42.4	1.00(0.45)		-0.698	0.201	12.07	<0.001
	Junior college or vocational high school	14.4	1.11(0.50)		-0.677	0.253	7.185	0.007
	High School	8.52	0.97(0.46)		-0.966	0.282	11.755	<0.001
	Junior College	8.71	1.10(0.57)		-0.839	0.288	8.474	0.004
	College and above	4.59	0.88(0.55)		-0.812	0.361	5.070	0.024
Monthly income				<0.001				
	(No income)	18.54	1.18(0.45)		–	–	–	–
	< ¥2000	5.81	1.28(0.39)		0.565	0.402	1.978	0.160
	¥2001–4000	25.19	1.18(0.46)		-0.046	0.221	0.043	0.836
	¥4001–7000	23.03	1.09(0.48)		-0.098	0.222	0.198	0.657
	> ¥7000	27.43	0.98(0.51)		-0.568	0.213	7.081	0.008

Percent (%): Percentage of total sample size.

M(SD):Means and standard deviations of subthreshold depression scores.

*p(t/F)*: Significance of results of t-test and F-test.

“( )”: Reference group for binary logistic regression.

β: Coefficients of the binary logistic regression model.

Standard error:In binary logistic regression, standard errors are usually associated with the model coefficients (β) and are used to assess the stability of the β estimates.

Wald x^2^: Testing the significance of the effect of a single independent variable on subthreshold depression.

*P*: Significance of the results of binary logistic regression.

The degree of subthreshold depression was measured using the CES-D, with a cutoff score of 16 being considered to indicate a likely depressive state, based on the recommendations of the scale’s developer (58). CES-D scores were used to create a binary variable (≥ 16 marked as 1 and < 16 marked as 0), and binary logistic regression analyses were performed for gender, HIV status, education level, and monthly income. The data fit (i.e., predictive accuracy) was 70.9%. As **Table 1** shows, women are more likely to develop subthreshold depression than men. No significant differences were found between participants who were HIV-negative and those who were HIV-positive (p = 0.120). Education level and monthly income were significant influences on subthreshold depression. Compared to participants who received a primary school education or lower, more highly educated participants were less likely to show symptoms of subthreshold depression. Participants with no income were more likely to show symptoms of subthreshold depression compared to participants with a monthly income of 7,000 yuan or more.

**Table 2** shows the means in the HIV-negative and HIV-positive groups for age, education level, and monthly income in each dimension and whether the differences in the results are significant. The results showed that age had gender (p = 0.002) and group differences (p < 0.001). Men had higher levels of education (p < 0.001) and monthly income (p = 0.017) than women, while participants in the HIV-positive group had higher levels of education and monthly income (p < 0.001) than those in the HIV-negative group. Gender (p < 0.001) and group (p < 0.001) differences were also found for perceived social support from significant others, friends, and family, with men receiving more support than women, and participants in the HIV-positive group receiving more support than those in the HIV-negative group. Regarding the dimensions of the CES-D, gender and group differences were found for negative affect, with women exhibiting more pronounced negative affect than men (p < 0.001). Gender differences were also found for somatic complaints (p < 0.001), with women scoring significantly higher than men. Interpersonal difficulties showed significant gender (p < 0.001) and group differences (p = 0.002). For DSTS scores, stereotype awareness (p = 0.006), stereotype identification (p < 0.001), and alienation (p < 0.001) showed gender differences, with higher scores for men, while group differences were found for stereotype identification (p < 0.001), as the HIV-positive group had significantly higher scores than the HIV-negative group.

**Table 2:** Gender and group differences in relevant scale dimensions (N = 1068).

	HIV-negative group M (SD)(n = 895)		HIV-positive group M (SD)(n = 173)		*p*	
	Male	Female	Male	Female	*p*(gender)	*p*(group)
	(n = 384)	(n = 511)	(n = 164)	(n = 9)		
Age	40.10(9.45)	41.01(10.35)	37.12(9.63)	44.00(4.42)	0.002	<0.001
Education level	2.32(1.29)	2.44(1.19)	3.41(1.78)	2.78(1.29)	<0.001	<0.001
Monthly income	2.58(1.55)	2.03(1.31)	2.89(1.33)	1.56(1.88)	0.017	<0.001
Significant other support	4.91(1.42)	4.29(1.53)	5.24(1.49)	5.86(1.04)	<0.001	<0.001
Friend support	4.89(1.44)	4.38(1.53)	4.79(1.42)	4.94(1.53)	<0.001	<0.001
Family support	4.33(1.33)	4.00(1.33)	5.17(1.46)	5.31(1.33)	<0.001	<0.001
Negative affect	0.77(0.58)	1.14(0.67)	0.91(0.67)	0.89(0.83)	<0.001	0.007
Positive affect	1.81(0.76)	1.62(0.71)	1.53(0.86)	1.97(0.71)	0.043	<0.001
Somatic complaints	0.83(0.58)	1.06(0.63)	0.88(0.60)	0.76(0.74)	<0.001	0.073
Interpersonal difficulties	0.68(0.76)	1.03(0.86)	0.66(0.75)	0.83(1.27)	<0.001	0.002
Self-appraisal	2.63(0.55)	2.59(0.59)	2.58(0.61)	2.19(0.76)	0.310	0.297
Self-acceptance	2.32(0.52)	2.30(0.56)	2.39(0.50)	1.90(0.67)	0.094	0.163
Stereotype awareness	3.10(1.13)	2.92(0.97)	3.13(1.12)	3.33(1.29)	0.006	0.095
Stereotype identification	3.65(1.23)	3.24(1.01)	3.75(0.98)	3.78(0.97)	<0.001	<0.001
Alienation	3.25(0.94)	2.93(0.97)	3.16(0.99)	3.56(097)	<0.001	0.151

P(gender): “gender” means that all participants were grouped by gender, P(group): “group” means that all participants were grouped by whether they whether they were HIV-positive or not.

### Correlation analysis

**Figure 1:**
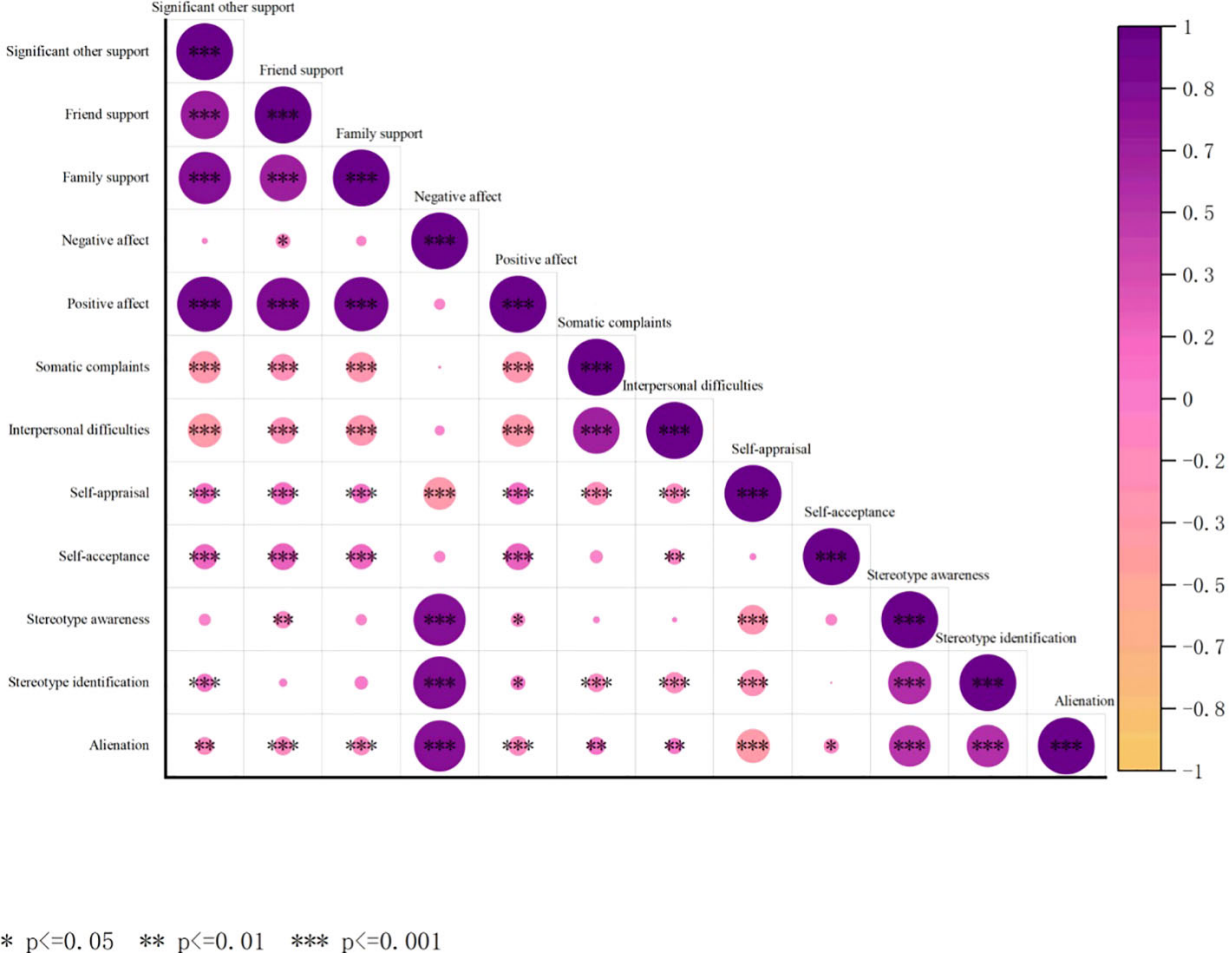
Heatmap plot of correlations between perceived social support, subthreshold depression, self-acceptance, and stereotype threat among people with substance use disorders.

**Figure 1** shows the correlations between the study variables. The three dimensions of perceived social support were significantly negatively correlated with interpersonal difficulties and significantly positively correlated with positive affect. Self-appraisal and self-acceptance were significantly and positively correlated with the three dimensions of perceived social support. Self-appraisal was significantly negatively correlated with negative emotions, somatic complaints and interpersonal difficulties. Drug use stereotype threat also showed positive correlations with some of the dimensions in subthreshold depression. Self-appraisal showed negative correlations with all three dimensions of drug use stereotype threat.

### Linear regression analysis

**Table 3** shows the relationships among the dimensions of the PSSS, SAQ, and DSTS and subthreshold depression after controlling for HIV status, gender, education level, and monthly income. This table shows the beta coefficients and standard errors in a regression model in which the dimensions are regressed on the CES-D controlling for four demographic variables. The results showed that self-appraisal, stereotype identification, and alienation were significantly associated with subthreshold depression among men, while significant other support, friend support, stereotype identification, and alienation were significantly correlated with subthreshold depression among women.

**Table 3:** Relationships between PSSS, SAQ, and DSTS scores and subthreshold depression in male and female participants.

Characteristics		Male (n = 548)			Female (n = 520)	
	β(SD)	t	*P*	β(SD)	t	*P*
Perceived social support						
Significant others support	-0.037(0.023)	-1.629	0.104	-0.118(0.025)	-4.739	<0.001
Friend support	-0.035(0.018)	-1.917	0.056	0.081(0.021)	3.793	<0.001
Family support	-0.024(0.021)	-1.168	0.243	-0.028(0.024)	-1.134	0.257
Self-acceptance						
Self-appraisal	-0.255(0.034)	-7.534	<0.001	-0.052(0.035)	-1.497	0.135
Self-acceptance	-0.051(0.035)	-1.471	0.142	-0.042(0.035)	-1.201	0.230
Drug use stereotype threat						
Stereotype awareness	0.012(0.20)	0.600	0.549	0.003(0.026)	0.137	0.891
Stereotype identification	-0.048(0.018)	-2.586	0.010	-0.120(0.028)	-4.258	<0.001
Alienation	0.094(0.023)	4.004	<0.001	0.075(0.026)	2.850	0.005

### Structural equation modeling

**Figure 2:**
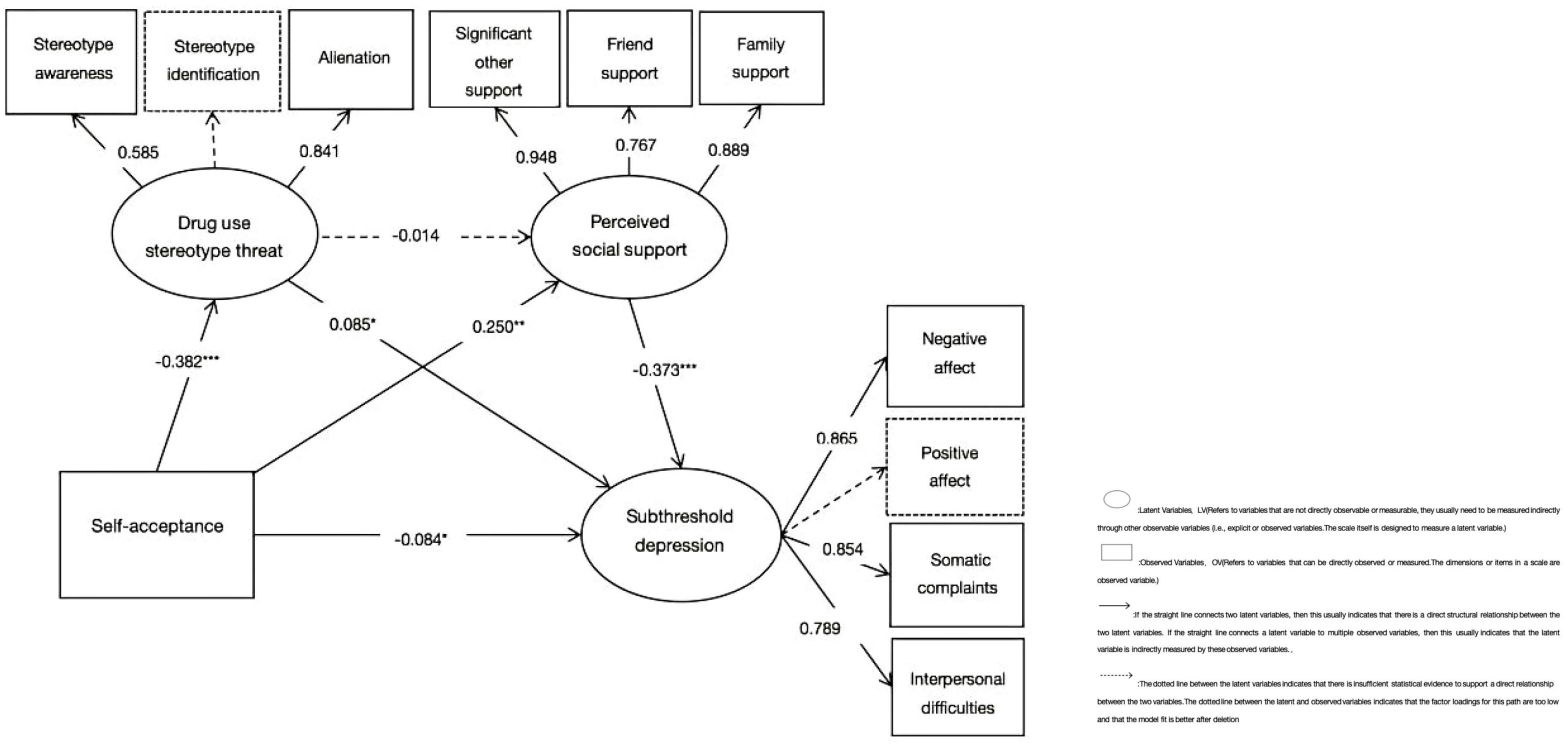
Model 1 of the mediating roles of drug use stereotype threat and perceived social support. *p < 0.05, **p < 0.01, ***p < 0.001.

In this model, the total mean score of the two dimensions of self-acceptance self-appraisal represented participants’ level of self-acceptance. First, we found a significant direct effect of the predictor variable (SAQ) on the dependent variable (subthreshold depression) in the unmediated model (β = -0.340, p < 0.001). Accordingly, we built Model 1 (**Figure 2**) with drug use stereotype threat (M1) and perceived social support (M2) as mediators, and bootstrap analyses with bias correction (2000 samples) showed that most paths were significant.

Here, the dimensions of stereotype awareness and positive affect were deleted because the factor loadings were too low (0.271/0.311) and some of the paths were more significant after deletion with better model fit. This structural equation model fit the data well, x2/df = 3.289, NFI = 0.986, CFI = 0.990, RFI = 0.974, RMSEA = 0.046. In this model, self-acceptance was negatively correlated with subthreshold depression (β = −0.084, p = 0.011, 95%CI [-0.157, -0.015]) and stereotype threat (β = -0.382, p < 0.001, 95%CI [-0.449, -0.305]), and positively correlated with perceived social support (β = 0.250, p = 0.002, 95%CI [0.181, 0.310]). The correlation between stereotype threat and perceived social support was not significant (β = −0.014, p = 0.721, 95%CI [-0.101, 0.063])but was positively correlated with subthreshold depression (β = 0.085, p = 0.035, 95%CI = [0.006, 0.165]). Perceived social support was negatively associated with subthreshold depression (β = −0.373, p < 0.001, 95%CI [-0.431, -0.309]). Stereotype threat (M1) partially mediated the association between self-acceptance and subthreshold depression (indirect effect: β = -0.046, p = 0.029, 95%CI [-0.095, -0.004], accounting for 15.23% of the total effect). Perceived social support (M2) was found to partially mediate the relationship between self-acceptance and subthreshold depression (indirect effect: β = -0.133, p = 0.002, 95%CI [-0.174, -0.093], accounting for 44.04% of the total effect; total effect: β = -0.302, p < 0.001, 95%CI [-0.395, -0.213]). However, M1 and M2 were not significant chain mediators between self-acceptance and subthreshold depression (β = -0.003, p = 0.710, 95%CI [-0.022,0.013]).

**Figure 3:**
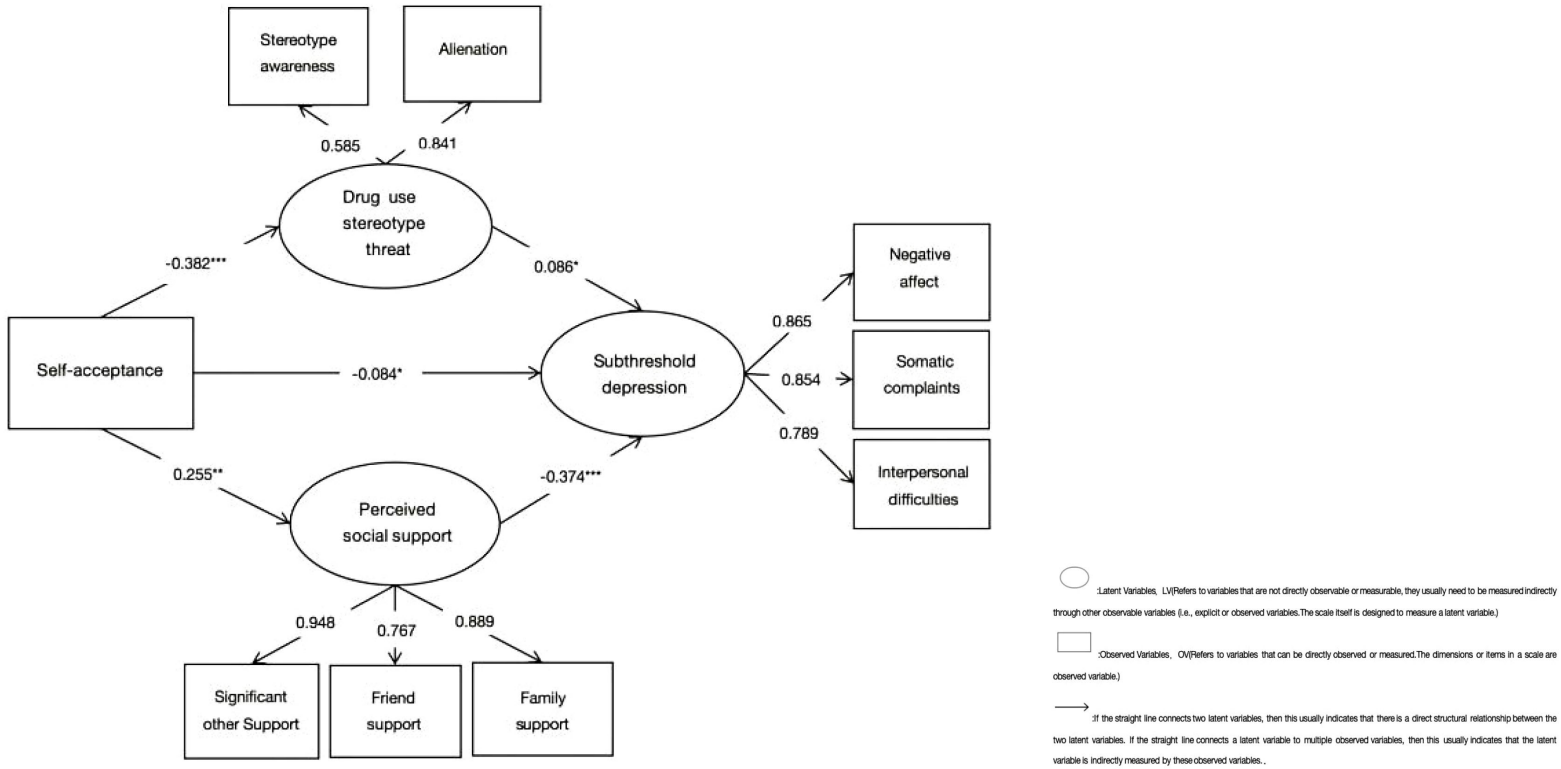
Model 2 of the parallel mediation of drug use stereotype threat and perceived social support. *p < 0.05, **p < 0.01, ***p < 0.001.

The parallel mediator model, Model 2 (**Figure 3**), was a modified version of Model 1. The model fit the data well, x2/df = 3.318, NFI = 0.986, CFI = 0.990, RFI = 0.975, RMSEA = 0.045. Self-acceptance was negatively associated with stereotype threat (β = -0.382, p < 0.001, 95%CI [-0.449,-0.305]), while stereotype threat was negatively correlated with subthreshold depression (β = 0.086, p = 0.038, 95%CI [-0.006, 0.165]). Self-acceptance was positively related to perceived social support (β = 0.255, p = 0.002, 95%CI [0.192, 0.312]), and perceived social support was positively correlated with subthreshold depression (β = -0.374, p < 0.001, 95%CI [-0.432, -0.310]). Self-acceptance was negatively correlated with subthreshold depression (β = -0.084, p = 0.011, 95%CI [-0.157, -0.016]). Drug use stereotype threat (M1) partially mediated the relationship between self-acceptance and subthreshold depression (indirect effect: β = -0.047, p = 0.032, 95%CI [-0.094, -0.005], accounting for 15.56% of the total effect). Perceived social support (M2) partially mediated the relationship between self-acceptance and subthreshold depression (indirect effect: β = -0.136, p < 0.001, 95%CI [-0.176, -0.098], accounting for 45.03% of the total effect; total effect: β = -0.302, p < 0.001, 95%CI [-0.395, -0.213]). The indirect effects (95% confidence intervals) of Models 1 and 2 are shown in **Table 4**.

**Table 4:** Indirect effects of Models 1 and 2 (95% confidence intervals).

		Estimate	Bootstrap 95% CI		*p*
			Lower limit	Upper limit	
Model 1	self-acceptance→drug use stereotype threat	-0.382	-0.449	-0.305	< 0.001
	drug use stereotype threat→subthreshold depression	0.085	0.006	0.165	0.035
	self-acceptance→drug use stereotype threat→subthreshold depression	-0.046	-0.095	-0.004	0.029
	self-acceptance→perceived social support	0.250	0.181	0.310	0.002
	perceived social support →subthreshold depression	-0.373	-0.431	-0.309	< 0.001
	self-acceptance→perceived social support →subthreshold depression	-0.133	-0.174	-0.093	0.002
	self-acceptance→drug use stereotype threat→perceived social support→subthreshold depression	-0.003	-0.022	0.013	0.710
	drug use stereotype threat→perceived social support	-0.014	-0.101	0.063	0.721
	self-acceptance→subthreshold depression	-0.084	-0.157	-0.015	0.011
	self-acceptance→perceived social support	0.255	0.192	0.312	0.002
Model 2	self-acceptance→drug use stereotype threat	-0.382	-0.449	-0.305	< 0.001
	drug use stereotype threat→subthreshold depression	0.086	0.006	0.165	0.038
	self-acceptance→drug use stereotype threat→subthreshold depression	-0.047	-0.094	-0.005	0.032
	self-acceptance→perceived social support	0.255	0.192	0.312	0.002
	perceived social support →subthreshold depression	-0.374	-0.432	-0.310	< 0.001
	self-acceptance→perceived social support →subthreshold depression	-0.136	-0.176	-0.098	< 0.001

### Buffering effect of perceived social support between self-acceptance and subthreshold depression

We classified self-acceptance into three categories based on SAQ score percentiles: high self-acceptance (SAQ scores ≥75th percentile), moderate resilience (SAQ scores > 25th percentile and < 75th percentile), and low resilience (SAQ scores ≤ 25th percentile).

We also categorized PSSS scores into three categories: high social support (social support scores ≥ 75th percentile), moderate social support (social support scores > 25th percentile and < 75th percentile), and low social support (social support scores ≤ 25th percentile) (61). Line graphs were used to display the effects of social support on the relationship between self-acceptance and subthreshold depression in different groups.

**Figure 4:**
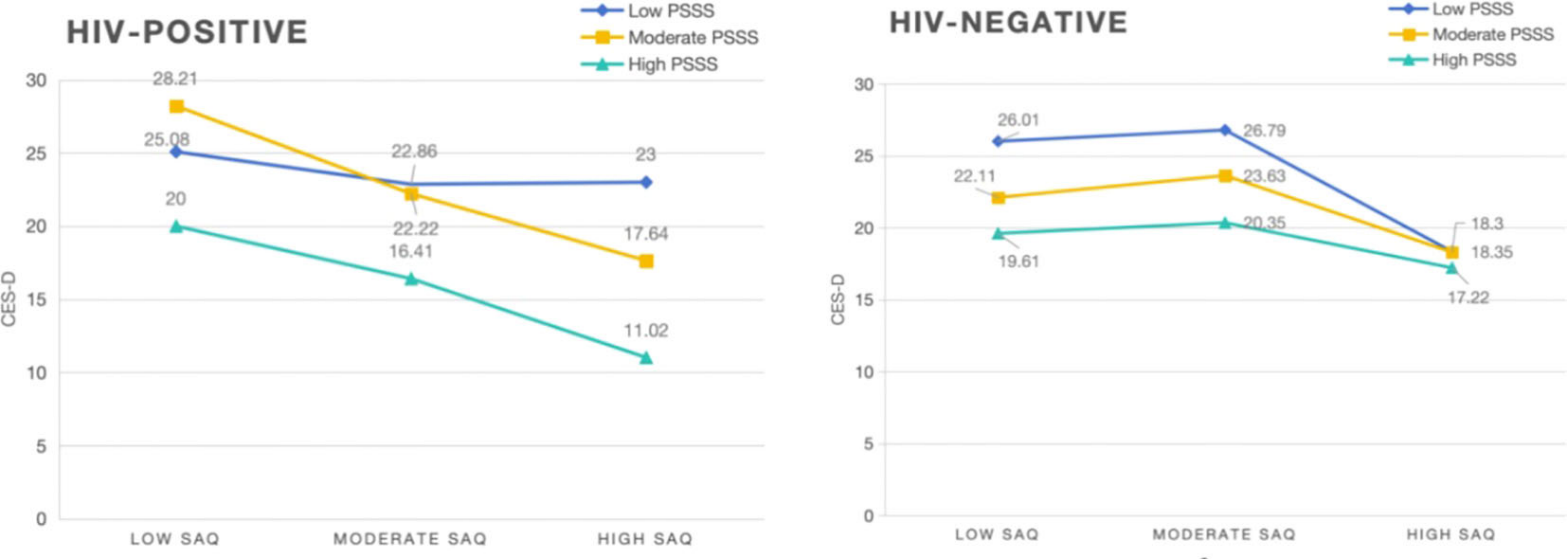
Buffering effect of perceived social support on the relationship between self-acceptance and subthreshold depression (HIV-negative: n = 895, HIV-positive: n = 173).

**Figure 4** shows that, among participants in the HIV-negative group with high levels of self-acceptance, low, moderate, and high levels of social support all had a buffering effect on subthreshold depression to some extent.

**Figure 5:**
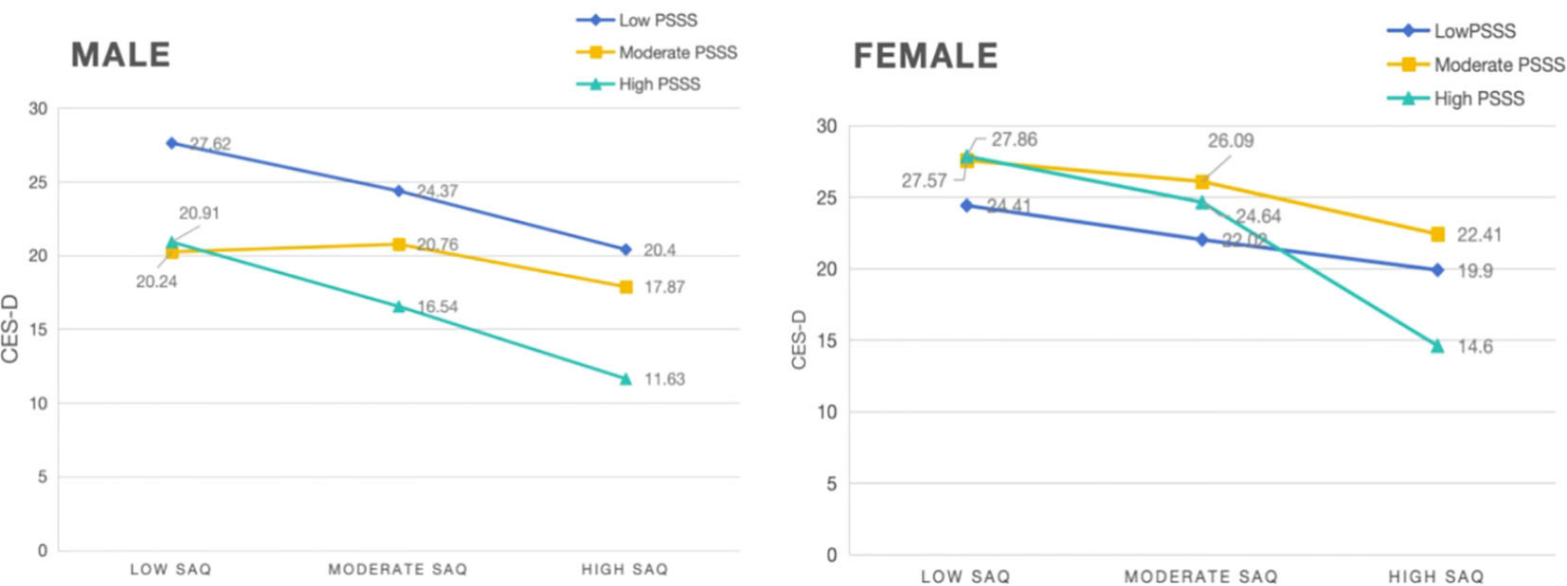
Buffering effect of perceived social support on the relationship between self-acceptance and subthreshold depression (male: n = 548, female: n = 520).

**Figure 5** shows that high levels of perceived social support had a significant buffering effect on subthreshold depression in female participants with high levels of self-acceptance.

## Discussion

In this study, women were found to be more at risk for developing subthreshold depression than men. Education and monthly income levels negatively affected subthreshold depression among people with SUD, while those who were HIV-positive had lower levels of subthreshold depression than those who were HIV-negative.

The World Health Organization has reported that the lifetime prevalence of depressive episodes in women is 3.2%, which is almost twice as high as that in men (62). Globally, women who use drugs face multiple health vulnerabilities, including poor mental health (63). This study found that women were significantly more likely to experience subthreshold depression than men, suggesting that more attention should be paid to the mental health of women within the population of people with SUD.

Furthermore, the results of this study revealed that both higher education levels and higher monthly incomes were associated with a lower risk of experiencing subthreshold depression. Consistent with the direction of previous findings, among those ≥ 20 years of age, higher education levels were associated with a lower prevalence of depressive symptoms (64). Cohort studies have demonstrated the long-term effects of educational level on anxiety and depression, with low educational attainment being significantly associated with both, and in longitudinal analyses, the protective effect of education was found to accumulate over time (65). The severity of depressive symptoms is negatively correlated with cognitive functioning, and since cognition is generally fostered by education, a negative correlation between education and depression may exist (66). We can infer that education can support and improve the psychological state of people with SUD at the cognitive level and that higher education levels have a positive effect on learning detoxification methods, improving cognitive styles, and reinforcing self-control, thereby resulting in reduced subthreshold depression prevalence through higher levels of education.

Research conducted in Korea has shown that poor job security, low income, and temporary/routine job type increase the risk of depressive symptoms 2–4 years after exposure (67). Studies have also indicated an association between a greater risk of being diagnosed for depression and insufficient food and monthly incomes below $600 (68). This is also true for people with SUD, as income is a significant risk factor for subthreshold depression. In addition, low monthly income may increase the risk of sexual dysfunction in people with major depressive disorder (69). Socioeconomic status may also be related to an increased risk of depression (70). As people with SUD face more negative stereotypes and discrimination, they are subjected to far greater difficulties and hindrances in finding employment, with consequences such as lower income, which may influence the less favorable overall situation of subthreshold depression among people with SUD.

While the depressive symptoms of people with SUD who are HIV-positive are associated with insufficient food and low income (68), the results of this study showed that participants with HIV had higher education levels, monthly income, and perceived social support than those who did not have HIV, which, in this context, explains the lower level of subthreshold depression in people with SUD who are HIV-positive compared with those who are HIV-negative.

## Consistent with Hypothesis 1:

### Negative correlation between self-acceptance and subthreshold depression among SUD patients

Self-acceptance has an important influence on mental health, as people with higher levels of self-acceptance are usually more resilient when facing stress and adversity (71). Furthermore, a negative correlation has been proposed between self-acceptance and mental health problems such as anxiety and depression (72). Individuals with low levels of self-acceptance tend to experience more negative emotions, including symptoms of depression and anxiety, suggesting a protective effect of self-acceptance against mental health disorders (73). This is consistent with the findings of this study that subthreshold depression levels decrease as self-acceptance increases in people with SUD. Owing to their special characteristics, people with SUD may experience more negative life events (e.g., job-seeking discrimination, difficulties making friends) than the general population, reducing their self-acceptance and resulting in more negative emotions, which can trigger subthreshold depression.

## Consistent with Hypothesis 2 and 3, partially consistent with Hypothesis 4:

### Perceived social support and drug use stereotype threat mediate the relationship between self-acceptance and subthreshold depression, respectively, forming parallel mediating relationships

Huang et al. (72) found that self-acceptance and perceived social support were protective factors for mental health among incarcerated offenders in China. This indicates that higher levels of self-acceptance and perceived social support are associated with better mental health. The likelihood of subthreshold depression occurring may be reduced as a result. Huang et al. (72) also noted that self-acceptance can mediate the relationship between perceived social support and mental health, and the similarities between people with SUD and incarcerated offenders in compulsory drug treatment centers suggest that self-acceptance, perceived social support, and subthreshold depression in groups that use drugs may be closely linked. Drug addiction stigma is a crucial factor that exacerbates depression and anxiety symptoms (63). People who use drugs and are frequently labeled negatively by society and prone to stigma and discrimination tend to have lower self-acceptance. However, when social support is received from significant others, family, and friends, the negativity of this stigma and discrimination is diminished to some extent and subthreshold depression is less likely to develop.

Data from an ERP experiment conducted by Zeng et al. (74) showed that participants in the “addict-negative words” condition were more accurate and had slower reaction times than those in the “addict-positive words” condition, suggesting a clear negative group stereotype of people with SUD. Thus, the threat posed by such stereotypes negatively affects the mental health of people with SUD and may result in subthreshold depression, which is not conducive to their reintegration into society. Results from a previous baseline study showed that on days with higher status, participants reported higher self-esteem and lower depression, anxiety, and shame. On days with higher self-esteem, participants reported lower depression, anxiety, and shame (75). Therefore, when people with SUD can increase their levels of self-acceptance and improve their negative identities and low self-esteem, this inward identification will reduce external pressures. In turn, this will reduce the threat of stereotypes brought by the outside world, which is also conducive to mitigating the likelihood of the onset of subthreshold depression.

## Contrary to Hypothesis 5, consistent with Hypothesis 6:

### Buffering effect of perceived social support on subthreshold depression in high self-acceptance levels among female and HIV-negative people with SUD

Previous research has shown that social support may modulate risk behaviors among intravenous drug users and that a lack of social support from a special person or significant other is associated with depressive symptoms in both men and women (76). Available social support can reduce the risk of mental health problems such as depression and anxiety by improving an individual’s social relationships and increasing their coping resources (77). In a sample of veterans with a history of depression, family support was negatively associated with depression (78), and receiving more family support was associated with a lower likelihood of depression. Adults who have a lack of social support show more severe depressive symptoms (79). Emotional social support has been found to significantly and negatively predict later depressive symptoms (80), indicating that having adequate emotional social support may reduce the possibility of depression. Furthermore, perceived relationship quality may be a better predictor of loneliness, depression, and psychosocial stress compared with frequency of contact (81); thus, perceived social support shows a stronger relationship with the quality rather than the quantity of interactions. This study’s results indicate that social support has a significant buffering effect for people with SUD who are HIV-negative with high self-acceptance, suggesting that different levels of social support can help to reduce subthreshold depression in this population. This may be because self-acceptance has been shown to affect resilience and coping (59), as individuals with high self-acceptance tend to have higher levels of resilience. Social support is a protective factor in the relationship between the effects of stress and physical and mental health (82), as well as an important resilience factor that buffers the negative psychological effects of stressful life events, thereby boosting overall well-being and life satisfaction (83, 84). People with SUD who are HIV-negative and have high self-acceptance may subjectively further amplify the role of social support for a significant effect on their subthreshold depression levels due to their greater resilience.

Japanese research has shown that “acceptance” is an explanatory variable for psychological distress in middle-aged women (85). Self-acceptance attenuates psychological distress and contributes to reducing the potential for subthreshold depression to occur. Since social support is a protective factor for depression in the female population, satisfaction with access to social support is the most favorable predictor of depressive symptoms (77). Women with high levels of self-acceptance are less prone to subthreshold depression after receiving high levels of social support in response to a combination of subjective and objective factors, which demonstrates the buffering effect of perceived social support.

### Implications and limitations

Finally, it is important to note that the present study used binary logistic regression to explore subthreshold depression among people with SUD and to explore gender and group differences in scale-related dimensions among HIV-negative and HIV-positive people with SUD. We also found mediating effects of drug use stereotype threat and perceived social support, as well as moderating effects of perceived social support in male/female, HIV-negative/HIV-positive groups, enriching understanding of self-acceptance and the development of subthreshold depression in people with SUD and providing some guidance for future interventions.Several limitations must be considered when interpreting the results of this study. In addition, this study has the following limitations. First, this study was conducted with people with SUD and therefore cannot be compared with the general population. Second, the participants recruited for this study were residents of two drug rehabilitation centers in Sichuan Province, which may have limited the representativeness of the sample. Third, when categorizing HIV-positive and negative groups in the study, the sample size of female HIV-positive participants was too small, which may have had some impact on the results. Fourth, the cross-sectional design of this study was unable to capture individual psychological and emotional changes such as in self-acceptance and subthreshold depression.

## Conclusions

The present study provides a basis for identifying a significant negative correlation between self-acceptance and subthreshold depression among people with SUD and demonstrates the mediating roles of perceived social support and drug use stereotype threat. Certain amount of social support had a significant role in controlling subthreshold depression levels. Perceived social support as a protective factor was effective in reducing the occurrence likelihood of subthreshold depression, and drug use stereotype threat as a risk factor increased the possibility of subthreshold depression. Providing appropriate social support and reducing stereotype threat through the joint efforts of society and people with SUD themselves would have great significance for maintaining the mental health of people with SUD and promoting their successful reintegration into society. Several factors should be considered in future research.

## Data Availability

The original contributions presented in the study are included in the article/supplementary material. Further inquiries can be directed to the corresponding author.
